# The Validation and Cross-Cultural Adaptation of the Arabic Version of the Polycystic Ovary Syndrome Quality of Life Scale (PCOSQOL)

**DOI:** 10.3390/jcm15020607

**Published:** 2026-01-12

**Authors:** Layan Alwatban, Ayah Sayed, Raneem Alwatban, Mais Alwatban, Nada Alyousefi

**Affiliations:** 1 University Family Medicine Center, King Saud University Medical City (KSUMC), King Saud University (KSU), P.O. Box 7805, Riyadh 11472, Saudi Arabia; 2 College of Medicine, King Saud University, P.O. Box 2925, Riyadh 11461, Saudi Arabia; ayah.kh.sayed@gmail.com (A.S.); raneemalwatban@gmail.com (R.A.); 3 College of Medicine, Princess Nourah Bint Abdulrahman University, P.O. Box 84428, Riyadh 11671, Saudi Arabia; maisalwatban@gmail.com; 4 Department of Family and Community Medicine, College of Medicine, King Saud University (KSU), P.O. Box 2925, Riyadh 11461, Saudi Arabia; nalyousefi@ksu.edu.sa

**Keywords:** polycystic ovary syndrome (PCOS), quality of life (QoL), PCOSQOL, Arabic translation, validation, Saudi Arabia

## Abstract

**Background:** Polycystic Ovary Syndrome (PCOS) is a common endocrine disorder, with a prevalence of approximately 16% in Saudi Arabia. PCOS is associated with various health complications. Assessing the quality of life (QoL) of women with PCOS is crucial for effective management. **Objectives:** This study aims to translate and validate the Polycystic Ovary Syndrome Quality of Life scale (PCOSQOL) into Arabic for use among Arabic-speaking women. The study was designed to evaluate the psychometric properties of the Arabic PCOSQOL, including its reliability, validity, and responsiveness. **Methods:** A cross-sectional study was conducted among 207 Saudi women diagnosed with PCOS. Participants were recruited from family medicine and obstetrics and gynecology clinics at King Saud University Medical City, Riyadh, through an online survey. The PCOSQOL was translated into Arabic following the World Health Organization’s (WHO) forward–backward translation protocol. Psychometric evaluation included internal consistency, test–retest reliability (ICC), and construct validity. **Results:** The Arabic PCOSQOL demonstrated excellent psychometric performance, with high internal consistency (Cronbach’s α = 0.951) and good-to-excellent test–retest reliability (ICC = 0.760–0.885). Construct validity was supported by a four-factor structure explaining 62.5% of the total variance (KMO = 0.92; Bartlett’s *p* < 0.001). The subscales showed strong factor loadings (0.49–0.97). Older women (>25 years), married participants, and residents of the western and central regions reported significantly better quality of life (*p* < 0.05). **Conclusions:** The Arabic version of the PCOSQOL demonstrated excellent reliability, validity, and stability, confirming its suitability for assessing quality of life among Arabic-speaking women with PCOS. This validated tool can support both clinical practice and future research across Arabic populations.

## 1. Introduction

Polycystic Ovary Syndrome (PCOS) is a prevalent endocrine disorder affecting women of reproductive age. Characterized by hyperandrogenism and ovulatory dysfunction, PCOS is associated with a spectrum of health complications. While various diagnostic criteria exist, the Rotterdam criteria, endorsed by the National Institute of Health (NIH) in 2012, remain widely accepted [1]. Hyperandrogenisms/hyperandrogenemia, and/or oligo/amenorrhea/anovulation, and/or polycystic ovarian morphology are the three Rotterdam criteria. Two criteria or more are needed for diagnosis, making a total of 4 phenotypes. In Saudi Arabia, PCOS affects approximately 16% of women and has a global prevalence of 11.5% based on Rotterdam criteria [2]. Significant prevalence underscores the importance of addressing PCOS-related health concerns in the region. Women with PCOS face increased risks for obesity, insulin resistance, type 2 diabetes, dyslipidemia, cardiovascular disease, nonalcoholic fatty liver disease, and obstructive sleep apnea. Moreover, they are more likely to experience mental health challenges such as depression and anxiety [3].

Accurate assessment of quality of life (QoL) is essential for understanding the multifaceted burden of PCOS and for guiding patient-centered management. Recent international guidance emphasizes that assessment of health-related quality of life (HRQoL) is a core component of PCOS care and research, reflecting the substantial psychosocial and emotional burden of the syndrome [4]. While several instruments—such as the PCOSQ and mPCOSQ—exist to evaluate PCOS-related QoL, their applicability in non-English-speaking contexts remains limited due to linguistic and cultural differences [5,6]. A culturally adapted and psychometrically sound Arabic tool is necessary to ensure that the unique concerns and experiences of Arab women are appropriately captured.

Recent Saudi data further highlight the impact of PCOS on health-related quality of life among affected women supporting the need for validated, context-appropriate patient-reported outcome measures (PROMs) in local clinical and research settings [7]. An Arabic PCOS-specific HRQoL instrument (AR-PCOSQ) has been translated and validated previously; however, AR-PCOSQ is a different measure from PCOSQOL in item content and domain structure. Therefore, an independent translation and psychometric validation of PCOSQOL in Arabic remains needed [8].

The Polycystic Ovary Syndrome Quality of Life scale (PCOSQOL), developed by Williams et al. (2018), is a 35-item, PCOS-specific instrument encompassing four key domains: Impact of PCOS, Infertility, Hirsutism, and Mood [9]. Its development involved patient-defined priorities, making it a robust candidate for cross-cultural adaptation. However, no validated Arabic version is currently available, despite the high prevalence of PCOS in Arab populations. Although PCOS has shared biomedical features across populations, the way symptoms translate into perceived quality of life is shaped by cultural norms, expectations, and social roles (e.g., societal meanings of fertility, appearance-related concerns such as hirsutism, and emotional well-being). Therefore, cross-cultural adaptation is not intended to change the clinical construct of PCOS, but to ensure that questionnaire items preserve their intended meaning and are interpreted consistently in the target language and culture, minimizing measurement bias and improving validity of comparisons across settings.

This study therefore aimed to translate, culturally adapt, and validate the PCOSQOL for use among Saudi women, in accordance with the World Health Organization’s (WHO) guidelines for tool translation. Establishing its reliability and validity will provide clinicians and researchers in the Arab world with a standardized measure for assessing PCOS-related quality of life in both clinical and research settings and inform personalized treatment plans.

## 2. Materials and Methods

### 2.1. Study Design

This study employed a cross-sectional design to translate and validate PCOSQOL for the use in Arabic-speaking populations, specifically among women with PCOS in Saudi Arabia.

### 2.2. Target Population

This study was conducted among Saudi women, Arabic speakers of childbearing age who were diagnosed with PCOS using the Rotterdam criteria and who attend family medicine clinics and/or obstetrics and gynecology clinics at King Saud University Medical City, Riyadh.

### 2.3. Sample Size with Sample Size Calculation/Sampling Technique

The following formula is used to estimate the sample size for this study: $n=Z^{2}\times p\times \left(1-p\right)/E^{2}$. Here, *n* = required sample size, Z = Z-value (the number of standard deviations from the mean, corresponding to the desired confidence level; for a 95% confidence level = 1.96), *p* = estimated prevalence of the condition (16% or 0.16), Margin of Error (E): Typically set at 5% or 0.05 [2]. *n* = (1.96)^2^ × 0.16 × (1 − 0.16)/(0.05)^2^ ≈206.24. We aimed for a minimum of 207 women diagnosed with PCOS to ensure sufficient statistical power for validation.

### 2.4. Subject Recruitment Procedures

Women diagnosed with PCOS, were recruited according to the inclusion criteria, who attended family medicine clinics and/or obstetrics and gynecology clinics at King Saud University Medical City, Riyadh.

### 2.5. Study Subject Selection

Inclusion Criteria: Arabic-speaking, Saudi women aged 18–45 years, who were diagnosed with PCOS based on Rotterdam criteria. Exclusion Criteria: All non-childbearing females, women pregnant at the time of data collection, women with diabetes before the diagnosis of PCOS, women with other significant endocrine disorders (adrenal dysfunction, thyroid dysfunction, and hyperprolactinemia), and those who have undergone surgical interventions affecting reproductive health (e.g., oophorectomy).

### 2.6. Study Setting

An online questionnaire survey was distributed to eligible participants via WhatsApp and was redistributed and filled a second time after 2–4 weeks. A 2–4 week interval is commonly used in test–retest studies to reduce recall effects while assuming no major clinical changes. Participants were instructed to complete the second administration under stable circumstances (i.e., without major treatment changes when possible) to allow assessment of measurement stability rather than true change in HRQoL.

### 2.7. Data Collection Tool

PCOSQOL [9] was used in addition to collecting data on demographic characteristics such as age, nationality, mother language, level of education, marital status, residence, medical diseases, surgical history, and pregnancy status. PCOSQOL is a self-administered questionnaire originally developed in English by Sophie Williams et al. [9]. The questionnaire contains a 35-item scale with four subscales (Impact of PCOS: how PCOS affects various areas of life, Infertility: assesses the emotional and psychological impact of infertility related to PCOS; Hirsutism: evaluates the impact of excessive hair growth; Mood: the emotional well-being and mood of women with PCOS), using a 7-point Likert-type scale ranging from ‘Does Not Apply’ (7) to ‘Usually’ (1). Lower scores represent a decreased QoL [9]. The PCOSQOL has shown good test–retest reliability, high internal consistency, and initial validity evidence, making it a useful tool for clinical and research settings.

### 2.8. Study Variables

Independent variables: Demographic characteristics such as age, nationality, mother language, level of education, marital status, residence, medical diseases, surgical history, and pregnancy status. Dependent variables: Impact of PCOS, infertility, hirsutism, and mood.

### 2.9. Study Procedure

Although Arabic PCOS-specific HRQoL questionnaires have been published, they are not equivalent to PCOSQOL in content and structure. PCOSQOL is a distinct instrument with its own item set and domain coverage (including mood-related items) and, to our knowledge, no validated Arabic PCOSQOL version was available. Therefore, translation and cross-cultural adaptation were required to enable accurate use of PCOSQOL in Arabic-speaking populations and to support comparability with studies using the original PCOSQOL. Questionnaire translation followed WHO’s guidelines for tool translation, which consists of 4 steps: forward translation, expert panel and back translation, pre-testing and cognitive interviewing, and the final version [10].

1. Forward translation: Two independent bilingual translators translated the original English PCOSQOL into Arabic, ensuring that medical and cultural contexts are considered.
2. Expert panel and back-translation: Two other bilingual experts back-translated the Arabic version into English to ensure accuracy and cultural appropriateness. After forward translation, a panel of five experts (two family physicians, two gynecologists and obstetricians, and a medical researcher with experience in questionnaire translation) revised it and compared it to the original English version. All are bilingual. Discrepancies between the original English version and the back-translated versions were discussed and resolved in a consensus meeting involving all translators.
3. Pre-testing and cognitive interviewing: The Arabic PCOSQOL was administered to a small group (20–30 participants) of women with PCOS, and feedback on clarity, cultural relevance, and any difficulties encountered was collected. Based on feedback, all necessary questionnaire modifications were made. These responses were not included in further analyses.
4. Final version: The Arabic version was subjected to validity and reliability testing. The tool was distributed to the target participants, where they completed the Arabic PCOSQOL, along with demographics and the required clinical information.

Although the forward–back translation and expert reconciliation minimized major discrepancies, a few issues required careful resolution to ensure conceptual equivalence in Arabic. In particular, some subjective terms (e.g., levels of “bother,” “embarrassment,” or emotional impact) were refined to preserve the intended intensity rather than relying on literal wording. We also prioritized Modern Standard Arabic phrasing that remains understandable across Arabic-speaking contexts while maintaining readability. Response-option anchors were reviewed to ensure equivalent gradations and consistent directionality. These steps help avoid common pitfalls in future translations, such as overly literal phrasing, region-specific terms, and mismatched Likert anchor meanings.

In this study, psychometric reliability refers to the consistency and stability of the instrument, assessed through internal consistency through Cronbach’s α and test–retest reliability through intraclass correlation coefficient (ICC). Cultural adequacy refers to achieving semantic, idiomatic, experiential, and conceptual equivalence between the original and translated versions, ensuring that items are understood as intended and remain relevant within the target cultural context.

### 2.10. Confidentiality and Ethical Consideration

Institutional Review Board (IRB) approval was obtained. The informed consent was explicit and indicated the purpose of the study and the right of the participant to withdraw at any time without any obligation towards the study team. No incentives or rewards were given to the participants. The gathered data was stored with a password only the researchers can access.

### 2.11. Statistical Analysis

Statistical analyses were conducted using SPSS (Group 1994) version 28 (IBM, Armonk, NY, USA). Continuous variables were summarized using mean ± SD, and categorical variables using frequency (%). For subgroup comparisons, age was dichotomized at the sample median (25 years) to create two similarly sized groups (≤25 vs. >25 years). Group differences were examined using independent-samples *t*-tests (two groups) or one-way ANOVA (multiple groups), as appropriate. Internal consistency was assessed using Cronbach’s α. Test–retest reliability was evaluated using ICC. Construct validity was examined using exploratory factor analysis (EFA). A two-tailed *p*-value < 0.05 was considered statistically significant.

## 3. Results

### 3.1. Overall Sample Characteristics

A total of 207 women with PCOS completed the questionnaire, with a mean age of 27.32 ± 6.86 years, and a median of 25 years old, where 104 participants were ≤25 years and 103 participants were >25 years. The majority held a bachelor’s degree (66.7%), were single (64.7%), and resided in the central region (87%). Nearly half (44.4%) reported monthly income below 5000 SAR. Employment status was distributed across students (34.3%), employed individuals (38.6%), and unemployed participants (26.1%). Approximately one-quarter (25.6%) of participants had children. Demographic characteristics are summarized in Table 1.

### 3.2. PCOSQOL Score Overview

The PCOSQOL uses a 7-point Likert scale where lower scores indicate poorer quality of life (greater negative impact) and higher scores indicate better quality of life (lesser or no impact). This scoring direction applies to all subscales and the total score.

Among all participants, the Impact of PCOS subscale yielded the highest mean score (72.85 ± 23.52), followed by Infertility (37.34 ± 12.00), Hirsutism (23.43 ± 11.65), and Mood (20.09 ± 9.06). The total mean PCOSQOL score was 153.71 ± 43.09. Overall PCOSQOL results are summarized in Table 2.

### 3.3. Psychometric Properties

#### 3.3.1. Internal Consistency Reliability

We first examined internal consistency reliability using Cronbach’s alpha to evaluate how well items within each subscale measured the same underlying construct. The Hirsutism, Infertility, and Impact of PCOS subscales all demonstrated excellent internal consistency (α = 0.943, 0.933, and 0.951, respectively), exceeding the α > 0.9 threshold for excellent reliability. The Mood subscale showed good reliability (α = 0.867), surpassing the α > 0.8 benchmark for acceptable reliability. The overall PCOSQOL exhibited excellent internal consistency (α = 0.951). These findings confirm that the Arabic PCOSQOL reliably measures each intended subscale. These results are summarized in Table 3.

#### 3.3.2. Construct Validity

Next, we assessed construct validity through EFA to determine whether the scale’s structure aligned with its theoretical framework. The analysis revealed excellent sampling adequacy (Kaiser–Meyer–Olkin [KMO] = 0.92), and Bartlett’s test of sphericity confirmed the data’s suitability for factor analysis (*p* < 0.001). Using greater than 1 eigenvalue rule, four factors were retained indicating a four-factor solution that adequately summarize the item correlation, collectively explaining 62.45% of the total variance—indicating good representation of quality-of-life constructs affected by PCOS.

**Figure 1:**
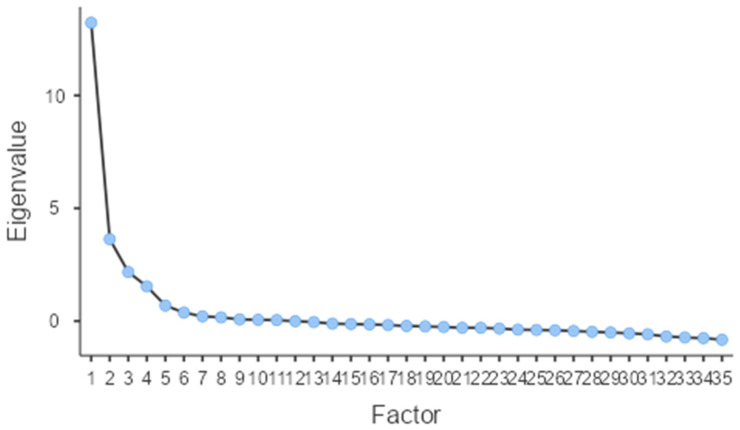
Scree Plot for the Eigenvalue of the extracted factors.

The four extracted factors corresponded to the original PCOSQOL subscales: Impact of PCOS (Factor 1, eigenvalue = 13.23, 25.11% variance), Infertility (Factor 2, eigenvalue = 3.63, 13.99% variance), Hirsutism (Factor 3, eigenvalue = 2.17, 13.82% variance), and Mood (Factor 4, eigenvalue = 1.54, 9.51% variance). All 35 items demonstrated strong factor loadings on their theoretically intended factors, ranging from 0.49 to 0.97, with no significant cross-loadings observed. Model fit indices showed acceptable fit (RMSEA = 0.08, 90% CI [0.08, 0.09]; TLI = 0.85), meeting the RMSEA < 0.08 criterion for acceptable fit. The four-factor solution replicated the original structure, supporting cross-cultural validity of the Arabic PCOSQOL. Factor loadings are shown in Table 4 and Figure 1.

#### 3.3.3. Test–Retest Reliability

We then evaluated test–retest reliability to assess the stability of PCOSQOL scores over time using ICC analysis with 50 participants who completed the questionnaire at two different time points. Scores were highly stable across administrations, with ICCs ranging from 0.760 (Mood) to 0.885 (Hirsutism). Specifically, the Hirsutism (ICC = 0.885), Infertility (ICC = 0.830), Impact of PCOS (ICC = 0.849), and overall score (ICC = 0.861) all demonstrated good to excellent reliability according to Koo & Li (2016) criteria [11], with values approaching or within the excellent range (>0.90). The Mood subscale showed good reliability (ICC = 0.760). All ICCs were statistically significant (*p* < 0.001) and exceeded the 0.75 threshold for good reliability. This stability suggests the tool can reliably monitor quality of life over time in clinical and research settings. ICCs are shown in Table 5.

### 3.4. Group Comparisons

Finally, we examined whether PCOSQOL scores differed across demographic and clinical subgroups to understand factors associated with quality of life in PCOS. Group comparisons across demographic and clinical subgroups for all PCOSQOL domains are presented in Table 6.

#### 3.4.1. Hirsutism

Older participants (>25 years) reported significantly better outcomes than younger participants (*p* = 0.007). Married women showed better quality of life related to hirsutism compared to single women (*p* = 0.005). Employed and unemployed participants reported better outcomes than students (*p* = 0.014). Participants with children showed higher scores than those without children (*p* = 0.004). See Table 6.

#### 3.4.2. Infertility

Younger participants (≤25 years) reported significantly better quality of life regarding infertility concerns than older participants (*p* < 0.001). Single women showed higher scores than married women (*p* < 0.001). Students reported better outcomes than employed and unemployed participants (*p* = 0.002). Participants without children showed higher scores than those with children (*p* < 0.001). See Table 6.

#### 3.4.3. Mood

Only marital status showed significant association with mood-related quality of life (*p* = 0.005), with married participants reporting better outcomes than single and divorced/widowed individuals. See Table 6.

#### 3.4.4. Impact of PCOS

Older participants (>25 years) reported significantly better overall quality of life than younger participants (*p* = 0.028). Regional differences were significant (*p* = 0.014), with western and central region residents reporting better outcomes than southern region residents; however, interpretation of regional differences should be made cautiously because participants were predominantly from the central region, and the numbers from other regions were small.

No significant differences were found across educational levels or income categories for any subscale.

## 4. Discussion

### 4.1. Psychometrics

An appropriate cultural adaptation and validation positively affect how patients from a specific culture perceive a health-related questionnaire. Language barriers can further affect the outcomes of research or patient care using a questionnaire with a non-native language. This study is the first—to our knowledge—to validate the PCOSQOL in Arabic using WHO translation methodology, a sufficiently powered sample (n = 207), and comprehensive psychometric testing (internal consistency, EFA, ICC).

Given the high prevalence of PCOS, with a local prevalence of 16% in Saudi Arabia [2], and the limited availability of validated Arabic PCOS quality-of-life instruments, this study addressed an essential gap. The available ones have limited validity for including only two PCOS phenotypes and the lack of an appropriate QoL scale based on the World Health Organization Quality of Life (WHOQOL) Group’s (1994) QoL proposal [6]. Moreover, the inconsistency of the main QoL domains between PCOSQoL-42 and PCOSQoL-47 mandates validating another PCOS-related QoL questionnaire for the benefit of both research and clinical settings [12,13,14]. Hence, adapting the PCOSQOL that is grounded in the WHOQOL framework and reflecting domains identified by affected women will offer an opportunity to establish a psychometrically sound, culturally appropriate instrument for use in Arabic-speaking populations.

A self-administered questionnaire for measuring HRQoL in women with PCOS was developed by Cronin et al. and published in 1998 with a total of 26 items, including five domains (emotions, body hair, weight, infertility, and menstrual problems) [5]. PCOSQ was translated into many languages, including Arabic, by Alghadeer et al., where they resulted to be reliable, valid, and culturally acceptable [8,15,16,17,18]. However, validity in the UK was affected due to the absence of an acne scale [19]. Barnard et al. modified PCOSQ by adding an acne scale [6]. mPCOSQ was translated into Chinese, Urdu, and Dutch and found reliable and valid [20,21,22]. Both scales showed good reliability and validity with the limitation of the narrow inclusion criteria of recruiting women with only hyperandrogenism and menstrual disturbance, which suggests that important aspects of women with PCOS that can impact quality of life may have been overlooked or excluded from the development of the scale.

An Arabic health-related quality-of-life questionnaire for married and unmarried women with PCOS (PCOSQoL-47 and PCOSQoL-42, respectively) was developed and published in 2021. PCOSQoL-47 consists of 5 domains (psychological and emotional status, fertility and sexual life, body image, hair disorder and acne, and obesity and menstrual disorder), whereas PCOSQoL-42 consists of 5 similar domains except for sexual health and obesity and the addition of the coping domain (psychological and emotional status, menstrual disorders and fertility, body image, hair disorder and acne, and coping). The inconsistency of the main QoL domains between PCOSQoL-42 and PCOSQoL-47 with a response rate of 8.6:1, was considered a limitation that resulted in a reduction in the validity [12,13,14].

Sophie Williams et al. have developed and preliminarily validated “a PCOS-specific QoL measure” which encompasses areas of QoL defined as “important by women with the condition” [9]. PCOSQOL was published in 2018 as a self-administered questionnaire consisting of a 35-item scale with four subscales (Impact of PCOS, Infertility, Hirsutism, and Mood), taking into consideration all phenotypes of PCOS based on Rotterdam criteria [23]. PCOSQOL is more sensitive, as it reflects PCOS’s psychological, social, and environmental domains, which are crucial in developing a QoL scale. PCOSQOL showed good reliability and is preliminarily validated. It provides broader relevance and stronger psychometric grounding.

Our Arabic version demonstrated internal consistency (Cronbach’s α = 0.951) comparable to that reported in prior studies, including the original English version [9,22,24,25]. The four extracted factors replicated the theoretical model—Impact of PCOS, Infertility, Hirsutism, and Mood—with high factor loadings (0.49–0.97), supporting cross-cultural validity. In contrast to the Dutch translation of PCOSQOL, where 6 items had loaded on a fifth factor, “coping with PCOS,” that was originally in the “Impact of PCOS,” and there was a failure to obtain 0.50 for item 20 [22]. As for the Indian translation, 3 factors have loaded (impact of PCOS, hirsutism, and infertility) as 0.22, 0.12, and 0.10, respectively, leading to a final 25-item PCOSQOL-I scale [25]. Additionally, the excellent sampling adequacy (KMO = 0.92) and explained variance (62.5%) underscore the structural robustness of the Arabic version.

Similarly to previous validations—such as the Dutch and Pashto translations—test–retest reliability was high (ICC = 0.760–0.885), confirming the stability of the scale over time [22,24]. These results strengthen confidence in the instrument’s reproducibility for both research and clinical use. In contrast, the PCOSQOL-I scale demonstrated a limitation by not assessing test–retest stability [25].

The Arabic version was developed in Modern Standard Arabic (Fusha), which is expected to enhance cross-regional comprehensibility among Arabic-speaking populations.

### 4.2. Cultural and Psychosocial Interpretation

Beyond psychometric soundness, our subgroup analyses reveal culturally meaningful patterns in how Saudi women experience PCOS. Regarding QoL related to hirsutism, higher scores indicating better quality of life reported by our study participants were in married women, employed and unemployed older participants (>25 years), and participants with children. Married participants reported better outcomes for the mood subscale, which reflects social support and marital reassurance, which mitigate body image and emotional distress.

Younger participants (≤25 years), single women, students, and participants without children reported significantly better quality of life regarding infertility concerns, as this result could be attributed to the possibility of not trying to conceive in these groups. This pattern is consistent with findings from the UK validation study by Williams et al. [9]. Similar results were reported in the original PCOSQOL development study, showing significantly lower QoL scores in comparison to those who were not trying to conceive (t(193)  = −6.48, *p*  <  0.001) [9]. Older participants (>25 years) and western and central region residents reported significantly better quality of life.

In Arab societies, fertility is closely tied to femininity and social identity; thus, infertility-related distress can be magnified by cultural expectations regarding marriage and motherhood [26]. Similarly, concerns over hirsutism and body image may interact with beauty norms and stigma surrounding visible hair growth [27]. These sociocultural nuances highlight the importance of localized instruments that capture emotional and relational dimensions specific to Middle Eastern contexts.

Prior studies in Saudi Arabia have examined HRQoL in women with PCOS using available measures [8]; however, these studies did not validate the PCOSQOL in Arabic. Our contribution differs in that we focused on the cross-cultural adaptation and psychometric validation of PCOSQOL specifically, enabling its use as a standardized instrument in Arabic-speaking settings and facilitating comparison with international studies that employ PCOSQOL. The adaptation process (expert review and cognitive interviewing) aimed to optimize clarity and conceptual equivalence of the Arabic wording, rather than altering the underlying domains of the instrument.

### 4.3. Limitations

Data were self-reported through an online survey, which may introduce selection bias or limit generalizability to less digitally connected populations. The study was conducted at a single institution, where it recruited more participant from the central region; future research should include diverse clinical settings to confirm external validity. Regional subgroup comparisons were underpowered due to the small number of participants from the northern and southern regions; therefore, these findings should be considered exploratory. A further limitation is that PCOS symptoms and perceived quality of life may fluctuate over time; therefore, observed test–retest differences may partially reflect true clinical or psychosocial changes rather than measurement instability.

Additionally, this validation was conducted among Saudi women; therefore, cultural norms and contextual factors may shape symptom reporting and the relative impact of PCOS domains (e.g., body image, fertility-related concerns, or disclosure of sensitive symptoms). Although the instrument was translated into Modern Standard Arabic using a rigorous cross-cultural adaptation approach to maximize cross-regional comprehensibility, the current psychometric evidence reflects a Saudi context. Future studies should replicate these findings in other Arabic-speaking countries and formally evaluate cross-country measurement invariance to confirm that scores are comparable across different Arabic-speaking populations and dialect contexts.

### 4.4. Implications and Future Directions

The validated Arabic PCOSQOL provides clinicians and researchers with a culturally sensitive tool to assess the multidimensional impact of PCOS on women’s lives. It enables identification of domains most affected in Arabic-speaking populations, allowing targeted counselling, patient education, and outcome evaluation.

Future studies should test the tool’s longitudinal responsiveness to lifestyle or pharmacological interventions and explore psychometric invariance across different Arab countries. Incorporating qualitative feedback could also enhance understanding of local experience of distress and body image perception related to PCOS.

## 5. Conclusions

In summary, this study established the Arabic PCOSQOL as a reliable, valid, and stable instrument for evaluating quality of life among women with PCOS. Its psychometric robustness and cultural relevance make it suitable for routine clinical assessment and cross-cultural research, supporting patient-centred care and future intervention studies in Arabic-speaking populations.

## Figures and Tables

**Table 1:** Demographic data of the studied participants.

Item	Total Participants (n = 207)
Age (years)	27.32 ± 6.86
Educational level	
High school or less	43 (20.8%)
Bachelor’s degree	138 (66.7%)
Master’s degree or above	9 (4.3%)
Vocational/Technical Institute/Diploma	17 (8.2%)
Marital status	
Single	134 (64.7%)
Married	65 (31.4%)
Divorced/widowed	8 (3.9%)
Residence	
Eastern region	9 (4.3%)
Western region	9 (4.3%)
Northern region	4 (1.9%)
Southern region	5 (2.4%)
Central region	180 (87%)
Income (SAR)	
<5000	92 (44.4%)
5000–9999	52 (25.1%)
10,000–19,999	45 (21.7%)
≥20,000	18 (8.7%)
Employment status	
Student	71 (34.3%)
Unemployed	54 (26.1%)
Employed	80 (38.6%)
Retired	2 (1%)
Having children	53 (25.6%)

Numerical data are presented as mean ± SD, and categorical data are presented as frequency (%).

**Table 2:** PCOSQOL of the studied participants (n = 207).

Item	Usually (1)	Often (2)	Sometimes (3)	Occasionally (4)	Rarely (5)	Not at All (6)	Does Not Apply (7)
During the last four weeks, how often have you:							
Hirsutism							
Been worried about other people seeing your facial hair	69 (33.3%)	25 (12.1%)	25 (12.1%)	19 (9.2%)	15 (7.2%)	33 (15.9%)	21 (10.1%)
Felt moody because of your excess hair	53 (25.6%)	19 (9.2%)	27 (13%)	16 (7.7%)	27 (13%)	41 (19.8%)	24 (11.6%)
Felt depressed because of your hirsutism	37 (17.9%)	15 (7.2%)	21 (10.1%)	13 (6.3%)	36 (17.4%)	53 (25.6%)	32 (15.5%)
Spent a lot of time and energy removing excess hair	57 (27.5%)	31 (15%)	16 (7.7%)	19 (9.2%)	17 (8.2%)	45 (21.7%)	22 (10.6%)
Felt depressed because of your facial hair	36 (17.4%)	18 (8.7%)	16 (7.7%)	10 (4.8%)	24 (11.6%)	68 (32.9%)	35 (16.9%)
Felt embarrassed by your facial hair	62 (30%)	20 (9.7%)	19 (9.2%)	10 (4.8%)	17 (8.2%)	51 (24.6%)	28 (13.5%)
Score	23.43 ± 11.65						
Infertility							
Felt under pressure to have a child	21 (10.1%)	6 (2.9%)	12 (5.8%)	11 (5.3%)	10 (4.8%)	36 (17.4%)	111 (53.6%)
Felt anxious about conceiving a child	41 (19.8%)	12 (5.8%)	29 (14%)	20 (9.7%)	18 (8.7%)	35 (16.9%)	52 (25.1%)
Felt depressed over difficulties conceiving a child	19 (9.2%)	6 (2.9%)	13 (6.3%)	9 (4.3%)	13 (6.3%)	43 (20.8%)	104 (50.2%)
Felt depressed because of your infertility	15 (7.2%)	5 (2.4%)	9 (4.3%)	3 (1.4%)	16 (7.7%)	36 (17.4%)	123 (59.4%)
Felt depressed over your struggle to have children	21 (10.1%)	5 (2.4%)	9 (4.3%)	3 (1.4%)	13 (6.3%)	40 (19.3%)	116 (56%)
Felt like a failure because of your trouble conceiving	16 (7.7%)	5 (2.4%)	9 (4.3%)	4 (1.9%)	15 (7.2%)	37 (17.9%)	121 (58.5%)
Been scared that you may not have children	38 (18.4%)	15 (7.2%)	25 (12.1%)	22 (10.6%)	20 (9.7%)	40 (19.3%)	47 (22.7%)
Score	37.34 ± 12						
Mood							
Felt guilty for being overly aggressive towards a friend or family member	40 (19.3%)	17 (8.2%)	32 (15.5%)	26 (12.6%)	21 (10.1%)	50 (24.2%)	21 (10.1%)
Overreacted to a day-to-day occurrence because of PCOS	44 (21.3%)	31 (15%)	46 (22.2%)	22 (10.6%)	21 (10.1%)	30 (14.5%)	13 (6.3%)
Felt overwhelmed by your PCOS and depression	56 (27.1%)	27 (13%)	38 (18.4%)	20 (9.7%)	19 (9.2%)	31 (15%)	16 (7.7%)
Had a short temper with your close friends and/or family	57 (27.5%)	25 (12.1%)	40 (19.3%)	28 (13.5%)	18 (8.7%)	29 (14%)	10 (4.8%)
Felt like crying for no reason	88 (42.5%)	27 (13%)	36 (17.4%)	23 (11.1%)	12 (5.8%)	16 (7.7%)	5 (2.4%)
Felt depressed	52 (25.1%)	22 (10.6%)	36 (17.4%)	26 (12.6%)	26 (12.6%)	31 (15%)	14 (6.8%)
Score	20.09 ± 9.06						
Impact of PCOS							
Felt like you weren’t a real woman because of your PCOS	14 (6.8%)	15 (7.2%)	24 (11.6%)	8 (3.9%)	26 (12.6%)	87 (42%)	33 (15.9%)
Felt depressed about how PCOS has impacted your life	33 (15.9%)	11 (5.3%)	29 (14%)	30 (14.5%)	23 (11.1%)	58 (28%)	23 (11.1%)
Felt like less of a woman because of having PCOS	15 (7.2%)	12 (5.8%)	25 (12.1%)	16 (7.7%)	25 (12.1%)	86 (41.5%)	28 (13.5%)
Wanted to do something but haven’t because of your PCOS	25 (12.1%)	12 (5.8%)	20 (9.7%)	19 (9.2%)	21 (10.1%)	79 (38.2%)	31 (15%)
Felt abnormal because of your PCOS	22 (10.6%)	8 (3.9%)	34 (16.4%)	19 (9.2%)	20 (9.7%)	75 (36.2%)	29 (14%)
Felt like you don’t know what to do to help yourself	47 (22.7%)	19 (9.2%)	32 (15.5%)	15 (7.2%)	16 (7.7%)	59 (28.5%)	19 (9.2%)
Felt like you don’t know what to do to control your PCOS	62 (30%)	23 (11.1%)	29 (14%)	21 (10.1%)	24 (11.6%)	31 (15%)	17 (8.2%)
Felt like you hated yourself	27 (13%)	13 (6.3%)	26 (12.6%)	23 (11.1%)	25 (12.1%)	70 (33.8%)	23 (11.1%)
Felt like your PCOS is in control of your life	37 (17.9%)	17 (8.2%)	33 (15.9%)	20 (9.7%)	23 (11.1%)	59 (28.5%)	18 (8.7%)
Felt embarrassed about the way you look	38 (18.4%)	19 (9.2%)	15 (7.2%)	16 (7.7%)	31 (15%)	64 (30.9%)	24 (11.6%)
Felt angry that you have PCOS	33 (15.9%)	15 (7.2%)	30 (14.5%)	25 (12.1%)	24 (11.6%)	59 (28.5%)	21 (10.1%)
Been envious of women without PCOS	19 (9.2%)	10 (4.8%)	13 (6.3%)	14 (6.8%)	21 (10.1%)	91 (44%)	39 (18.8%)
Felt embarrassed about having PCOS	17 (8.2%)	10 (4.8%)	17 (8.2%)	18 (8.7%)	20 (9.7%)	91 (44%)	34 (16.4%)
Felt that it is unfair that you have PCOS	14 (6.8%)	7 (3.4%)	9 (4.3%)	16 (7.7%)	21 (10.1%)	98 (47.3%)	42 (20.3%)
Struggled to cope with your PCOS and your other condition(s)	45 (21.7%)	17 (8.2%)	39 (18.8%)	17 (8.2%)	17 (8.2%)	46 (22.2%)	26 (12.6%)
Wanted to take part in a social activity but haven’t because of your PCOS	14 (6.8%)	6 (2.9%)	9 (4.3%)	8 (3.9%)	18 (8.7%)	108 (52.2%)	44 (21.3%)
Score	72.85 ± 23.52						
Total score	153.71 ± 43.09						

Numerical data are presented as mean ± SD, and categorical data are presented as frequency (%). A low score represents a lower quality of life; a higher score represents a lesser to no impact on quality of life.

**Table 3:** Internal consistency of the PCOSQOL domains for total participants (n = 207).

Domain	Cronbach’s Alpha	Level of Reliability
Hirsutism	0.943	Excellent
Infertility	0.933	Excellent
Mood	0.867	Good
Impact of PCOS	0.951	Excellent
Total	0.951	Excellent

**Table 4:** Factor loadings from the exploratory factor analysis.

		Factors			
		Factor 1	Factor 2	Factor 3	Factor 4
		Impact of PCOS	Infertility	Hirsutism	Mood
	Eigenvalue	13.23	3.63	2.17	1.54
	% of Variance	25.11	13.99	13.82	9.51
	Cumulative %	25.11	39.11	52.93	62.45
Q	Factor Loadings				
7	Been worried about other people seeing your facial hair			0.84	
21	Felt moody because of your excess hair			0.87	
22	Felt depressed because of your hirsutism			0.78	
24	Spent a lot of time and energy removing excess hair			0.81	
26	Felt depressed because of your facial hair			0.82	
30	Felt embarrassed by your facial hair			0.9	
1	Felt under pressure to have a child		0.86		
8	Felt anxious about conceiving a child		0.55		
9	Felt depressed over difficulties conceiving a child		0.9		
23	Felt depressed because of your infertility		0.89		
25	Felt depressed over your struggle to have children		0.97		
31	Felt like a failure because of your trouble conceiving		0.94		
34	Been scared that you may not have children		0.49		
2	Felt guilty for being overly aggressive towards a friend or family member				0.68
30	Overreacted to a day-to-day occurrence because of PCOS				0.79
5	Felt overwhelmed by your PCOS and depression				0.68
11	Had a short temper with your close friends and/or family				0.77
12	Felt like crying for no reason				0.59
14	Felt depressed				0.5
4	Felt like you weren’t a real woman because of your PCOS	0.69			
6	Felt depressed about how PCOS has impacted your life	0.62			
10	Felt like less of a woman because of having PCOS	0.62			
13	Wanted to do something but haven’t because of your PCOS	0.8			
15	Felt abnormal because of your PCOS	0.83			
16	Felt like you don’t know what to do to help yourself	0.69			
17	Felt like you don’t know what to do to control your PCOS	0.64			
18	Felt like you hated yourself	0.69			
19	Felt like your PCOS is in control of your life	0.74			
20	Felt embarrassed about the way you look	0.8			
27	Felt angry that you have PCOS	0.71			
28	Been envious of women without PCOS	0.64			
29	Felt embarrassed about having PCOS	0.76			
32	Felt that it is unfair that you have PCOS	0.62			
33	Struggled to cope with your PCOS and your other condition(s)	0.75			
35	Wanted to take part in a social activity but haven’t because of your PCOS	0.63			

Note. ‘Maximum likelihood’ extraction method was used in combination with an ‘oblimin’ rotation. Bartlett’s Test of Sphericity: *p*-value < 0.001. Kaiser–Meyer–Olkin (KMO) Measure 0.92.

**Table 5:** Intraclass correlation coefficient of the PCOSQOL domains for 50 participants at two different times.

Domain	ICC (95% CI)	*p*-Value	Level of Agreement	Mean Test ± SD	Mean Retest ± SD
Hirsutism	0.885 (0.798 to 0.934)	<0.001	Almost perfect	23.54 ± 12.35	22.44 ± 12.35
Infertility	0.830 (0.702 to 0.903)	<0.001	Almost perfect	35.4 ± 11.63	33.86 ± 12.49
Mood	0.760 (0.579 to 0.863)	<0.001	Substantial	17.96 ± 8.21	19.76 ± 9.98
Impact of PCOS	0.849 (0.734 to 0.914)	<0.001	Almost perfect	69.66 ± 24.73	70.76 ± 25.1
Total	0.861 (0.755 to 0.921)	<0.001	Almost perfect	146.56 ± 41.85	146.82 ± 48.73

ICC: Intraclass correlation coefficient, Statistical significance at *p*-value < 0.05.

**Table 6:** Group comparisons of different domains for the studied participants (n = 207).

Item	Hirsutism Score	*p*-Value	Infertility Score	*p*-Value	Mood Score	*p*-Value	Impact of PCOS Score	*p*-Value
Age (years)								
≤25	21.28 ± 11.25	0.007	40.6 ± 9.32	<0.001	19.9 ± 9.11	0.765	69.28 ± 23.82	0.028
>25	25.6 ± 11.7		34.06 ± 13.46		20.28 ± 9.05		76.46 ± 22.77	
Educational level								
High school or less	21.98 ± 11.8	0.767	39.58 ± 8.46	0.070	18.65 ± 8.98	0.455	67.14 ± 22.98	0.243
Bachelor’s degree	23.64 ± 11.49		37.02 ± 12.25		20.14 ± 9.27		73.86 ± 23.11	
Master’s degree or above	23.67 ± 12.31		28.33 ± 18.81		23.11 ± 7.18		81.89 ± 22.64	
Vocational/Technical Institute/Diploma	25.29 ± 12.92		39.06 ± 11.8		21.71 ± 8.44		74.29 ± 27.63	
Marital status								
Single	21.6 ± 11.56 ^a^	0.005	41.63 ± 7.94 ^a^	<0.001	19.16 ± 8.85 ^a^	0.005	71.54 ± 23.46	0.413
Married	26.31 ± 11.37 ^b^		28.48 ± 13.92 ^b^		22.75 ± 8.95 ^b^		74.49 ± 24.47	
Divorced/widowed	30.75 ± 8.55 ^ab^		37.63 ± 13.52 ^ab^		14 ± 8.55 ^a^		81.38 ± 14.42	
Residence								
Eastern region	23.89 ± 14.73	0.826	30.56 ± 14.84	0.081	18.22 ± 10.1	0.347	59.78 ± 31.42 ^ab^	0.014
Western region	25.44 ± 12.2		43.11 ± 6.09		23.22 ± 10.08		81.11 ± 19.9 ^a^	
Northern region	26 ± 12.3		40.75 ± 7.5		25.75 ± 11.27		82 ± 26.7 ^ab^	
Southern region	28.4 ± 9.02		28.2 ± 14.11		15.2 ± 8.56		44 ± 27.45 ^b^	
Central region	23.11 ± 11.59		37.57 ± 11.92		20.04 ± 8.92		73.69 ± 22.55 ^a^	
Income (SAR)								
<5000	21.92 ± 11.57	0.172	39.05 ± 10.4	0.152	18.95 ± 9.56	0.152	69.29 ± 25.64	0.156
5000–9999	23.13 ± 12.53		35.12 ± 13.22		19.5 ± 8.09		73.1 ± 20.62	
10,000–19,999	26.62 ± 10.59		37.82 ± 12.13		22.2 ± 8.61		76.87 ± 22.21	
≥20,000	24 ± 11.28		33.83 ± 14.65		22.39 ± 9.6		80.28 ± 21.53	
Employment status								
Student	19.8 ± 10.68 ^a^	0.014	41.41 ± 7.45 ^a^	0.002	19.52 ± 8.65	0.840	69.28 ± 24.11	0.234
Unemployed	25.56 ± 11.75 ^b^		35.72 ± 12.3 ^b^		20.37 ± 9.63		71.89 ± 24.55	
Employed	25.15 ± 11.91 ^b^		35.16 ± 13.98 ^b^		20.3 ± 9.17		76.86 ± 22.08	
Retired	26 ± 5.66 ^ab^		24 ± 15.56 ^ab^		24.5 ± 7.78		65 ± 21.21	
Having children								
No	22.06 ± 11.47	0.004	39.79 ± 10.14	<0.001	19.75 ± 9.15	0.352	72.49 ± 23.41	0.706
Yes	27.4 ± 11.37		30.25 ± 14.09		21.09 ± 8.82		73.91 ± 24.03	

Numerical data are presented as mean ± SD, and categorical data are presented as frequency (%), with statistical significance at a *p*-value < 0.05. Different lower-case letters indicate significant difference in pairwise comparison. For all scales, a lower score indicates lower quality of life and a higher score indicates a lesser to no impact on quality of life.

## Data Availability

The datasets generated and analyzed during the current study are not publicly available due to privacy restrictions but are available from the corresponding author upon reasonable request.

## Abbreviations

The following abbreviations are used in this manuscript:

PCOS	Polycystic Ovary Syndrome
PCOSQOL	Polycystic Ovary Syndrome Quality of Life
QoL	Quality of Life
EFA	Exploratory factor analysis
ICC	Intraclass correlation coefficient
NIH	National Institute of Health
SPSS	Statistical package for the social science
KMO	Kaiser–Meyer–Olkin
HRQoL	Health-related quality of life
WHO	World Health Organization
IRB	Institutional Review Board
PROMs	Patient-reported outcome measures
